# Characteristics of meibomian gland dysfunction in patients with Stevens–Johnson syndrome

**DOI:** 10.1097/MD.0000000000016155

**Published:** 2019-06-28

**Authors:** Tina Shrestha, Hyun Sik Moon, Won Choi, Hyeon Jeong Yoon, Yong Sok Ji, Mayumi Ueta, Kyung Chul Yoon

**Affiliations:** a Department of Ophthalmology, Chonnam National University Medical School and Hospital, Gwangju, South Korea; b Department of Ophthalmology, Kathmandu University School of Medical Science, Dhulikhel Hospital, Kavre, Nepal; c Department of Frontier Medical Science and Technology for Ophthalmology, Kyoto Prefectural University of Medicine, Kyoto, Japan.

**Keywords:** meibomian gland dysfunction, ocular manifestation, risk factors, Stevens–Johnson syndrome

## Abstract

To evaluate the characteristics of meibomian gland dysfunction (MGD) in patients with Stevens–Johnson Syndrome (SJS) and investigate the risk factors for severe MGD.

Sixteen patients with a history of SJS were evaluated for MGD. To assess the SJS severity acute ocular involvement score (AOS), acute systemic involvement score (ASS), and chronic ocular manifestation score (COMS) were measured. Meibomian gland parameters were evaluated using meibomian gland dropout score (meiboscore - using a Keratograph 5 M), meibum expression score (MES), meibum quality score (MQS), and lid margin abnormality score (LMAS). Correlations between severity of meibomian gland parameters and degree of ocular and systemic involvement of SJS were analyzed. Risk factors for development of severe MGD were identified.

The patients’ mean age was 32.0 ± 14.3 years. Four patients were men and 12 were women. MGD had developed in 14 patients (87.5%). The meibomian gland parameters were significantly correlated with ocular and systemic degree of SJS as evaluated using AOS (*P* < .01), ASS (*P* < .01), and COMS (*P* < .01). Patients with severe MGD had a higher AOS (*P* < .01) and COMS (*P* = .02) values than those without severe MGD. On multivariate analysis, AOS higher than 2 was a significant risk factor for developing severe MGD (*P* = .03).

MGD was a common ocular manifestation with SJS patients. Severity of meibomian gland parameters was correlated with AOS, ASS, and COMS, and the presence of acute ocular complications was a risk factor for severe MGD in patients with SJS.

## 1 Introduction

Stevens–Johnson syndrome (SJS) is an acute, self-limiting, but sometimes life-threatening disease, involving the skin and mucous membranes, including ocular surface, oral cavity and genitals.^[1–5]^ It is characterized by inflammatory vesiculobullous reactions. The pathogenesis of SJS is unknown, but it appears to involve cell–mediated keratinocyte apoptosis via the Fas signaling cascade and granular release.^[6]^ SJS can be caused by exposure to certain medications, infection or malignancy.^[7]^ Ocular manifestations of SJS can be categorized into acute stage, including conjunctivitis and meibomitis, and chronic stage including meibomian gland dysfunction (MGD) and ocular surface keratinization.^[8]^

The meibomian glands are located in the tarsal plates and secrete lipids onto the outermost layer of the tear film, providing a smooth surface for the cornea by lubricating during blinking.^[9]^ MGD is a chronic eyelid disease with a multifactorial etiology, causes ocular irritation and ultimately affect quality of life. The disease is caused by obstruction of terminal ducts, with quantitative and qualitative changes in glandular secretion, resulting in alteration of the tear film.^[10–12]^ The risk factors of MGD can be categorized into ophthalmic factors (aniridia, chronic lens wear, chronic blepharitis, Demodex folliculorum-related ocular disease, and eyelid tattooing, etc.), systemic factors (aging, atopic dermatitis, Sjögren syndrome [SS], SJS, and cicatricial pemphigoid, etc.), and therapeutic factors (isotretinoin, antidepressant, antihistamines, antiandrogens, etc.).^[13–16]^

Acute ocular complications occur in 50% to 88% of SJS cases.^[8]^ For instance, 1 study found that meibomitis was present in more than half of patients with SJS.^[2]^ Such complications are caused by inflammation and loss of ocular surface epithelium, which results in keratinocyte apoptosis. Chronic ocular complications occur in 35% of SJS cases.^[8]^ They are characterized by persistent and prolonged ocular surface inflammation and ulceration. MGD in SJS is caused by squamous metaplasia of the meibomian gland orifices, with secondary inspissations and inflammation eventually leading to meibomian gland atrophy and dropout.^[17]^

One previous study using the keratograph showed almost total meibomian gland dropout in two patients with SJS.^[18]^ Another investigation reported that 66.7% of patients with SJS had severe meibomian gland dropout.^[19]^

However, the correlation between MGD and ocular and systemic manifestations in patients with SJS has not been studied, neither have the risk factors for MGD development been identified. Therefore, in the present study, we aimed to evaluate the correlation between MGD severity and degree of ocular and systemic involvement in patients with SJS, as well as identify the risk factors for MGD severity.

## 2 Methods

This was a retrospective study of patients with SJS who has presented to the Department of Ophthalmology, Chonnam National University Hospital between November 2015 and December 2017. The diagnosis of SJS was based on clinical features, namely high fever, serious mucocutaneous illness with skin eruptions and involvement of at least two mucosal sites. This study was approved by the Institutional Review Board of the Chonnam National University Hospital. Informed consents were obtained from all participants.

The eligibility criteria were as follows:

1. confirmed history of SJS,
2. treatment for ocular complications of SJS, and
3. evaluation of MGD by grading.

The exclusion criteria were

1. previous ocular diseases other than SJS,
2. eyelid abnormalities, and
3. history of ocular surgery or trauma in the 3 months prior to enrollment.

### 2.1 Ocular and systemic involvement of SJS

The diagnostic criteria for acute complications of SJS have been reported previously.^[20]^ Acute ocular involvement score (AOS) ranged from 0 to 3, depending on the involvement of the conjunctival hyperemia, pseudomembrane, and corneal epithelial erosions. An AOS ≥2 was defined as indicating severe acute ocular complications.^[20]^ Acute systemic involvement score (ASS) ranged from 0 to 16 and was evaluated on oral or genital erythema, degree of epidermal detachment, liver dysfunction, high fever, respiratory disturbance, total epidermal necrosis, anemia, elevated C-reactive protein, kidney dysfunction and pneumonia. Severe systemic involvement was defined as an ASS ≥8.^[20]^ Sotozono et al revised a new grading system for evaluating of chronic ocular manifestation score (COMS) in patients with SJS.^[21]^ It ranged from 0 to 39. The score involves three structures (cornea, conjunctiva, and eyelid) and takes into account 13 clinical signs of ocular complications each of which is graded from 0 to 3 depending on its severity. A COMS ≥13 was considered to indicate severe chronic ocular complications.^[21]^

### 2.2 Meibomian gland evaluation

Meibomian gland examination consisted of subjective (meibomian gland dropout) and objective factors (meibum expressibility, quality and assessment of lid margin). Meibomian gland dropout was detected using infrared images of the upper and lower lid meibomian glands which were captured using by Keratograph 5 M (Oculus GmbH, Wetzlar, Germany). Meibomian gland dropout was graded from 0 to 3, as follows: 0, no meibomian gland loss; 1, ≤33% of meibomian gland loss; 2, 33% to 67% meibomian gland loss; and 3, >67% meibomian gland loss.^[22]^ The scores of the upper and lower eyelids were added, to obtain a total meiboscore (ranging from 0–6). The meibography images were analyzed using Image J reported by Srinivasan et al^[22]^ Meibum expression score (MES) and meibum quality score (MQS) were assessed by applying digital pressure to the tarsal plate by observing meibum secretion level and by evaluating the meibum quality and the number of the expressed meibomian gland orifices within the central eighth lower eyelid, in conjunction with slit lamp biomicroscope. MES ranged from 0 to 3, as follows: 0 was all meibomian glands expressible; 1, 3–4 meibomian glands expressible; 2, 1-2 meibomian glands expressible; 3, no meibomian glands expressible.^[23]^ MQS ranged from 0 to 3, as follows: 0, clear fluid; 1, cloudy fluid; 2, cloudy particulate fluid; 3, inspissated toothpaste like fluid.^[23]^ Lid margin abnormality scoring (LMAS) ranged from 0 to 3, as follows: 0, irregular of lid margin; 1, vascular engorgement; 2 glandular orifices plugged; 3, anterior or posterior displacement of mucocutaneous junction.^[24]^ Severe MGD was diagnosed when the meiboscore was ≥4 or when the sum of the MES, MQS, and LMAS was ≥5.

### 2.3 Tear film and ocular surface evaluation

The Schirmer test was carried out for 5 minutes without anesthesia. The strips were inserted at the junction of the lateral and middle third of the lower eyelid and the amount of wetting of the strip was measured using the millimeter scale. To determine tear film break up time (TFBUT), the interval between a complete blink and the appearance of the first dry spot was recorded after the fluorescein instillation. TFBUT was measured three times, and the mean value was recorded. Keratoepitheliopathy was examined by staining the cornea with fluorescein and scoring the area and density of staining.^[25]^ The severity of keratoepitheliopathy was evaluated by multiplying the area score by the density score, and this was used as an index of the damage in the corneal surface. The staining area was graded on a numerical scale of 0 to 3, as follows, 0 no punctate staining, 1 ≤ one third punctate staining, 2 one-third to two-thirds punctate staining, and 3 ≥ two-thirds punctate staining. The staining density was also graded on a numerical scale of 0 to 3 as follows: 0 no punctate staining, 1 sparse density, 2 moderate density, 3 high density with overlapping lesions.^[25]^

### 2.4 Statistics

Statistical analysis was performed using SPSS 18.0 (SPSS Inc., Chicago, IL). Data are presented as mean ± standard deviation. The chi-square test was used to compare differences in incidence between the sexes. Correlations between SJS involvement and MGD parameters were assessed by using Spearman rho correlation test. The Mann-Whitney U test was used to compare various parameters between the severe and non-severe MGD groups. Univariate logistic regression analysis was used to investigate risk factors related to severe MGD in patients with SJS. Multivariate logistic regression analysis was performed variables with *P* value < .05 in univariate analysis. All *P* values < .05 were considered statistically significant.

## 3 Results

The mean age of patients was 32.0 ± 14.3 years, ranging from (18–53 years). Out of 16 patients, 12 were women and four were men. The mean values of ASS, AOS, and COMS were 5.0 ± 2.4, 1.7 ± 1.2, and 3.1 ± 9.9, respectively. The mean meiboscore, MES, MQS, and LMAS were 3.6 ± 1.9, 1.7 ± 1.1, 1.8 ± 0.9, and 1.6 ± 1.1, as shown in Table 1. The most common chronic ocular complications in eyelid was meibomian gland dysfunction with 14 patients (87.5%), while in corneal complication was superficial punctate keratinization with 12 patients (75.0%) and in conjunctival complication was hyperemia with 10 patients (62.5%) respectively as shown in Table 2. There were significant correlations between all meibomian gland parameters with ocular and systemic involvement patients with SJS (*P* < .01 in all cases) (Table 3). Patients with SJS who had severe MGD showed higher AOS (*P* < .01) and COMS (*P* = .02) values than those without severe MGD. On the other hands, age, gender, ASS, TFBUT, Schirmer test, and keratoepitheliopathy value did not differ significantly among the groups as shown in Table 4. Univariate logistic regression revealed that age (OR, 7.47, *P* < .01), gender (OR, 4.15, *P* = .04) and AOS (OR, 7.47, *P* < .01) were significant risk factors for severe MGD. Multivariate analysis revealed that only AOS (≥2 / < 2) (OR, 4.82, *P* = .02) was an independent risk factors for severe MGD in SJS (Table 5).

**Table 1:**
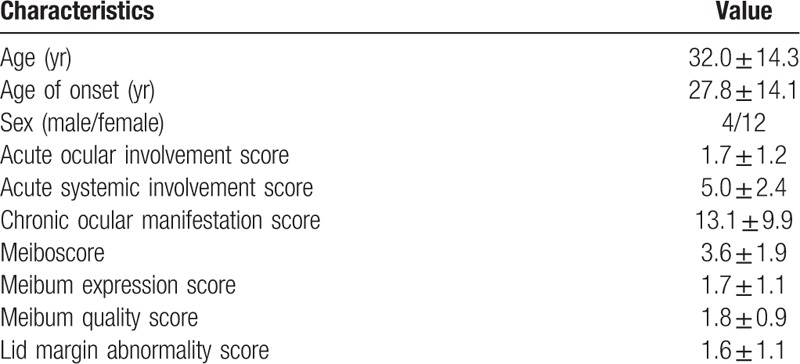
Clinical characteristics of patients with Stevens–Johnson syndrome.

**Table 2:**
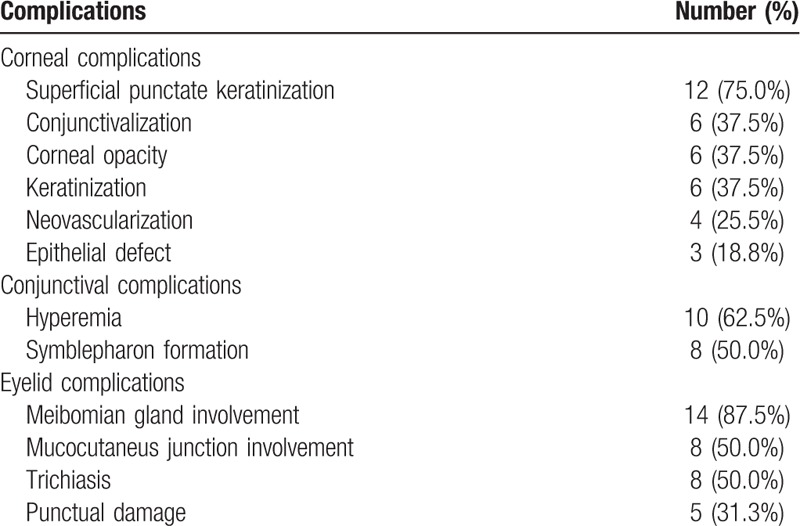
Chronic ocular complications in patients with Stevens–Johnson syndrome.

**Table 3:**
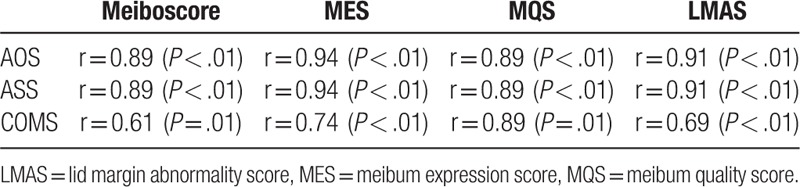
Correlations between severity of meibomian gland parameters and acute ocular involvement score (AOS), acute systemic involvement score (ASS) and chronic ocular manifestation score (COMS) in patients with Stevens–Johnson syndrome.

**Table 4:**
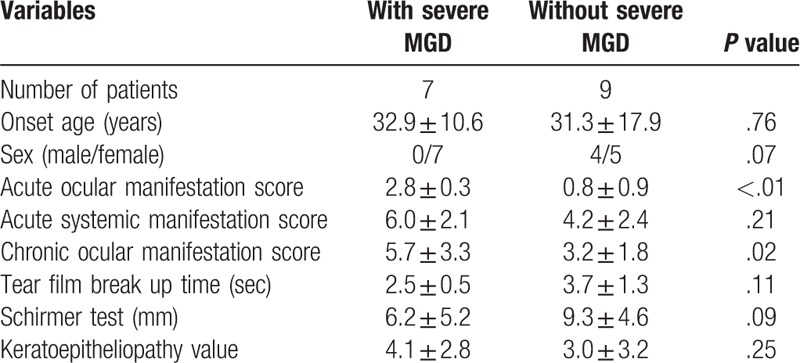
Comparison between patients with Stevens–Johnson syndrome who have severe meibomian gland dysfunction (MGD) and those who do not.

**Table 5:**
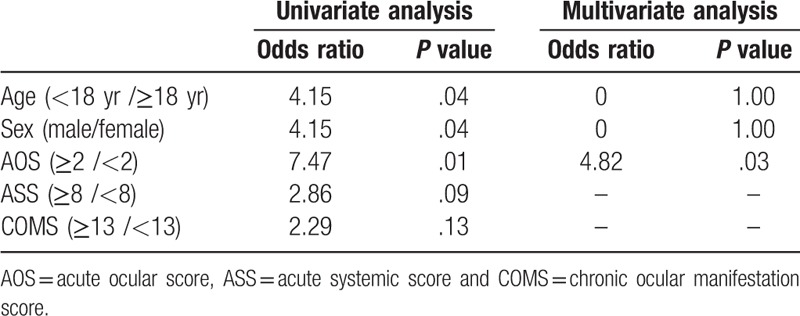
Risk factors for severe meibomian gland dysfunction in Stevens–Johnson syndrome.

## 4 Discussion

Recently Ueta carried out detailed investigations into the pathophysiology of SJS with severe ocular complications. Along with genetic predisposition, abnormal compromise of innate mucosal immunity was found to be the main reason that patients harbored commensal bacteria like methicillin-resistant staphylococcus aureus and methicillin-resistant staphylococcus epidermis, which cause severe ocular infection in SJS. Cold medicine and non-steroidal anti-inflammatory drugs thought to be a risk factor for SJS. However, the pathophysiological mechanism connecting cold medicines to SJS poorly understood, although multiple gene polymorphisms and their interactions contribute strongly. In this regard, genetic predispositions such as human leukocyte antigen (HLA) genotype differ among ethnicity. Specifically, HLA-A∗02:06 in the Japanese population and HLA-B∗44:03 among the Indian and Brazilian populations have been significantly associated with severe ocular complications in SJS. Investigations into single nucleotide polymorphisms have that toll-like receptor 3, EP3 protein of prostaglandin-E receptor 3, and Ikaros family zinc finger 1 (IKZF1) genes may contribute to ocular surface inflammation.^[26]^

SJS is one of the most important causes of ocular morbidity. It causes severe ulceration and inflammation and leads to scarring of the cornea, conjunctiva and eyelid, ultimately threatening vision itself. The correlation between MGD severity and ocular and systemic manifestations of SJS is poorly understood to date. In the present study, we elucidated the correlation between MGD severity and the degree of ocular and systemic SJS involvement; we then identified the risk factors for the development of severe MGD.

In the present study, among all chronic ocular complications of SJS, meibomian gland involvement was the most common (87.5%), corroborating previous studies that have shown a high rate of meibomian gland involvement in SJS.^[21,27]^ In SJS patients, eyelids tend to undergo cicatricial changes, and meibomian glands can be also damaged. Furthermore, the meibomian gland plays a critical role in stabilizing the tear film, and injury to it contributes to disruption of the tear film, causing severe dry eye.

Many other ocular surface diseases related to systemic diseases were associated with MGD. Patients with graft-versus-host disease (GVHD) showed meibomian gland destruction which correlated to the severity of disease as well as the grade of ocular surface damage.^[28]^ In addition, impairment of meibomian glands was more severe in dry eye patients with SS compared to non-SS dry eye.^[29]^ Kaido et al^[30]^ showed that functional visual acuity and clinical severity score of eyelids such as the MGD grade were markedly worsened in patients with SJS compared to the SS patients and control subjects.

The present study revealed that the severity of meibomian gland parameters (meiboscore, MES, MQS, and LMAS) was significantly correlated with ocular and systemic involvement of SJS (ASS, AOS, and COMS). Patients with SJS who had severe MGD showed higher AOS and COMS values than those without severe MGD. Among the various potential risk factors for severe MGD in patients with SJS, multivariate analysis showed that only AOS (≥2 / < 2) was an independent risk factor. As ASS indicates systemic involvement of SJS, it is not a risk factor for developing MGD. In previous reports the incidence of acute ocular involvement among hospitalized patients with SJS has been 27% to 80%.^[21]^ Another group reported that 75.3% of patients with SJS developed acute ocular complications.^[31]^

The cause of acute ocular involvement in SJS is highly active inflammation. Squamous metaplasia and inflammation of the meibomian gland in patients with SJS can eventually lead to meibomian gland atrophy and dropout.^[17]^ A reduction in the volume and quality of meibum secretion into the ocular surface causes alteration and thinning of the lipid layer, increasing tear film evaporation. This lack of lubrication aggravates blink-related micro-trauma to the cornea. If left untreated, this can induce squamous metaplasia, generating corneal pannus and finally leading to limbal stem cell deficiency, which affects the vision itself.^[32]^ Conservation treatments for MGD include lid hygiene, warm compression or physical treatment, and ocular lubricants including lid containing agents.^[33]^ If severe acute ocular involvements exist, intensive treatments for MGD such as anti-inflammatories or immunosuppressants, intense pulse light, and thermal pulsation therapy are needed to prevent chronic complications caused by micro-trauma associated with MGD.

The present study had several drawbacks. First, it was retrospective in nature and had a small sample size. Second, the etiology and treatment of SJS was not evaluated. In conclusion, present study showed that the prevalence of MGD among patients with SJS was 87.5%. The MGD severity was correlated with ocular and systemic involvement of SJS. The degree of acute ocular complications was a risk factor for developing severe MGD in SJS. We recommend that all patients in the acute stages of SJS be evaluated and closely followed by an ophthalmologist to monitor the occurrence of MGD.

## Acknowledgments

The authors thank Dr Punyaram Kharbuja, Department of Surgical Oncology, Bhaktapur Cancer Hospital, Nepal for his help in preparing the manuscript.

## Author contributions

**Conceptualization:** Kyung Chul Yoon.

**Data curation:** Hyeon Jeong Yoon.

**Investigation:** Tina Shrestha.

**Methodology:** Hyeon Jeong Yoon, Yong Sok Ji, Mayumi Ueta.

**Project administration:** Kyung Chul Yoon.

**Validation:** Won Choi.

**Writing – original draft:** Tina Shrestha.

**Writing – review & editing:** Hyun Sik Moon, Won Choi, Kyung Chul Yoon.
